# Dibutyl phthalate induced testicular dysgenesis originates after seminiferous cord formation in rats

**DOI:** 10.1038/s41598-017-02684-2

**Published:** 2017-05-31

**Authors:** Nathália L. M. Lara, Sander van den Driesche, Sheila Macpherson, Luiz R. França, Richard M. Sharpe

**Affiliations:** 1 MRC Centre for Reproductive Health, The Queen’s Medical Research Institute, University of Edinburgh, Edinburgh, EH16 4TJ UK; 2 Laboratory of Cellular Biology, Department of Morphology, Federal University of Minas Gerais, 31270-901 Belo Horizonte/MG, Brazil; 3 National Institute for Amazonian Research, 69067-375 Manaus/AM, Brazil; 4 Present Address: Centre for Integrative Physiology, Biomedical Sciences, Hugh Robson Building, University of Edinburgh, Edinburgh, EH8 9XD UK

**Keywords:** Developmental biology, Endocrinology

## Abstract

Administration of dibutyl phthalate (DBP) to pregnant rats causes reproductive disorders in male offspring, resulting from suppression of intratesticular testosterone, and is used as a model for human testicular dysgenesis syndrome (TDS). DBP exposure in pregnancy induces focal dysgenetic areas in fetal testes that appear between e19.5–e21.5, manifesting as focal aggregation of Leydig cells and ectopic Sertoli cells (SC). Our aim was to identify the origins of the ectopic SC. Time-mated female rats were administered 750 mg/kg/day DBP in three different time windows: full window (FW; e13.5–e20.5), masculinisation programming window (MPW; e15.5–e18.5), late window (LW; e19.5–e20.5). We show that DBP-MPW treatment produces more extensive and severe dysgenetic areas, with more ectopic SC and germ cells (GC) than DBP-FW treatment; DBP-LW induces no dysgenesis. Our findings demonstrate that ectopic SC do not differentiate de novo, but result from rupture of normally formed seminiferous cords beyond e20.5. The more severe testis dysgenesis in DBP-MPW animals may result from the presence of basally migrating GC and a weakened basal lamina, whereas GC migration was minimal in DBP-FW animals. Our findings provide the first evidence for how testicular dysgenesis can result after normal testis differentiation/development and may be relevant to understanding TDS in human patients.

## Introduction

The testicular dysgenesis syndrome (TDS) hypothesis has become a focus for research into the origins/causes of the commonest male reproductive disorders that manifest at birth (cryptorchidism, hypospadias) or in young adulthood (low sperm count, testis germ cell cancer)^1, 2^. The hypothesis proposes that incorrect ‘setting up’ of the testis during its normal differentiation leads to somatic (Sertoli, Leydig) cell dysfunction which results in TDS disorders; this is based on the observation that testes from men with a TDS disorder often show focal dysgenetic features in testis morphology^3–7^. From a developmental perspective, it is accepted that correct differentiation of the testis (as described below) is a pre-requisite for the next step which is phenotypic masculinisation, a process that is completely hormone-driven^8–10^. However, it is obviously impossible to prove in humans that morphological dysgenetic features in the testes of men with TDS disorders have arisen in fetal life as hypothesized, so the only recourse is to animal models^11^.

We have developed and validated an animal model for TDS, based on pregnancy exposure to the environmental chemical dibutyl phthalate (DBP)^12–14^. DBP exposure suppresses intratesticular testosterone in fetal males, resulting in TDS disorders^11, 15^, which are often accompanied by areas of focal dysgenesis in the testis, manifesting as malformed seminiferous tubules and abnormal distribution of somatic cells in these focal areas (e.g. intratubular Leydig cells)^12, 16, 17^. There are two big unsolved mysteries about the focal dysgenetic areas. First, the malformed seminiferous tubules are not evident until after birth^18^, so cannot be due to abnormal seminiferous cord formation, which occurs between embryonic day (e)13.5–e14.5 in rats^19–22^. Instead, the dysgenetic areas are thought to derive from focal areas of ectopic Sertoli cells found scattered in the interstitium of the fetal testis (i.e. outside of seminiferous cords) in DBP-exposed animals^12, 16–18, 23^. The second mystery is that the aforementioned ectopic Sertoli cells do not appear until beyond e19.5 in the rat^23^, long after the initial differentiation of Sertoli cells and formation of seminiferous cords. The latter events occur normally in DBP-exposed fetuses^18, 24^, but even when DBP exposure is initiated after cord formation (e15.5), ectopic Sertoli cells still appear later in gestation. Because of these observations, we have reasoned that either the ectopic Sertoli cells result from *de novo* differentiation between e19.5–e21.5 or they derive from the breakdown of already formed seminiferous cords. However, both of these scenarios are unprecedented and can be viewed as challenging the existing dogma about testis differentiation and development^25, 26^, outlined below.

Sertoli cells are the first somatic element to differentiate in the gonadal ridge and this event initiates testis differentiation and testis-specific gene expression^27–29^. The differentiating Sertoli cells then enclose the germ cells (gonocytes), which have migrated from the hindgut to the gonadal ridge^25, 30^. The ‘nests’ of Sertoli cells + gonocytes are then encircled by presumptive peritubular myoid cells to form the seminiferous cords, a process completed by e14.5 in rats^8, 18, 31^. After cord formation, the fetal Leydig cells differentiate and begin to produce insulin-like factor 3 and testosterone which, together with anti-müllerian hormone produced by the Sertoli cells, are responsible for masculinising the internal and external genitalia^8, 11^.

Testosterone is the most important of the hormones produced by the fetal testis, as it is responsible for masculinisation of the reproductive tract^8^. These effects are programmed by androgens in a critical time window (e15.5–e18.5 in rats), termed the ‘masculinisation programming window (MPW)’, which determines later reproductive tract development and final reproductive organ size^32–35^. Androgen deficiency during the MPW increases the incidence of TDS disorders and reduces the adult size of all male reproductive organs and anogenital distance (AGD)^32–36^. Moreover, DBP-induced androgen deficiency during the MPW induces later appearance of ectopic Sertoli cells at e21.5, an event that can be classed as dysgenesis.

The primary aim of the present study was to resolve the mystery outlined above, by elucidating the mechanistic origin of ectopic Sertoli cells in the testes of rats after DBP exposure. Integral to this, was a comparison of DBP exposure in different fetal time windows, to discover why only DBP exposure in the MPW results in ectopic Sertoli cells/dysgenesis, and why restricting DBP treatment to the MPW causes more severe dysgenesis than exposing rats to DBP for longer (but including the MPW).

## Results

### Focal testicular dysgenetic areas appear when DBP exposure occurs in the MPW

**Figure 1:**
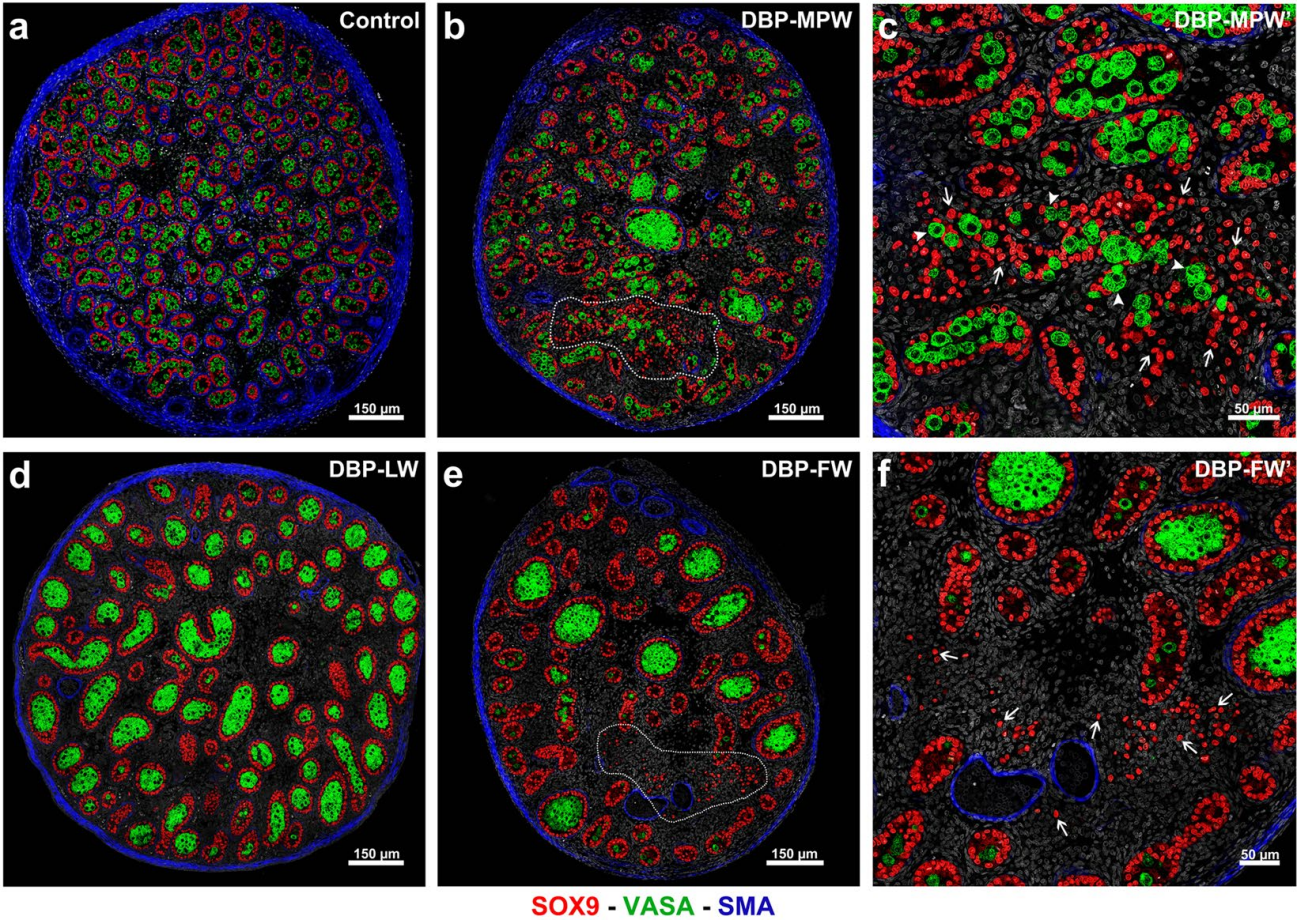
Effect of fetal exposure of rats to DBP during the masculinisation programming window (MPW), the late window (LW) or the full window (FW) on testis histology at e21.5. Sections were triple immunostained for SOX9 (red, Sertoli cells), VASA (green, germ cells), and smooth muscle actin (blue). When present (DBP-MPW and DBP-FW), focal dysgenetic areas are circled by a white dotted line (b, e). Note the presence of ectopic Sertoli (red) and germ (green) cells in the interstitial compartment.

Rats from DBP-MPW and DBP-FW groups both showed the appearance of focal dysgenetic areas at e21.5, in which ectopic Sertoli (SOX9+) and germ (VASA+) cells were evident in the interstitial compartment (Fig. 1b,c,e,f). These focal dysgenetic areas were not present in the testes of control or DBP-LW rats (Fig. 1a,d). Unexpectedly, the dysgenetic areas observed in the testes of DBP-MPW fetuses appeared to be more extensive than those found in DBP-FW fetuses (Fig. 1c,f and Supplementary Fig. S1).

### DBP exposure does not induce de novo differentiation of Sertoli cells outside the seminiferous cords

**Figure 2:**
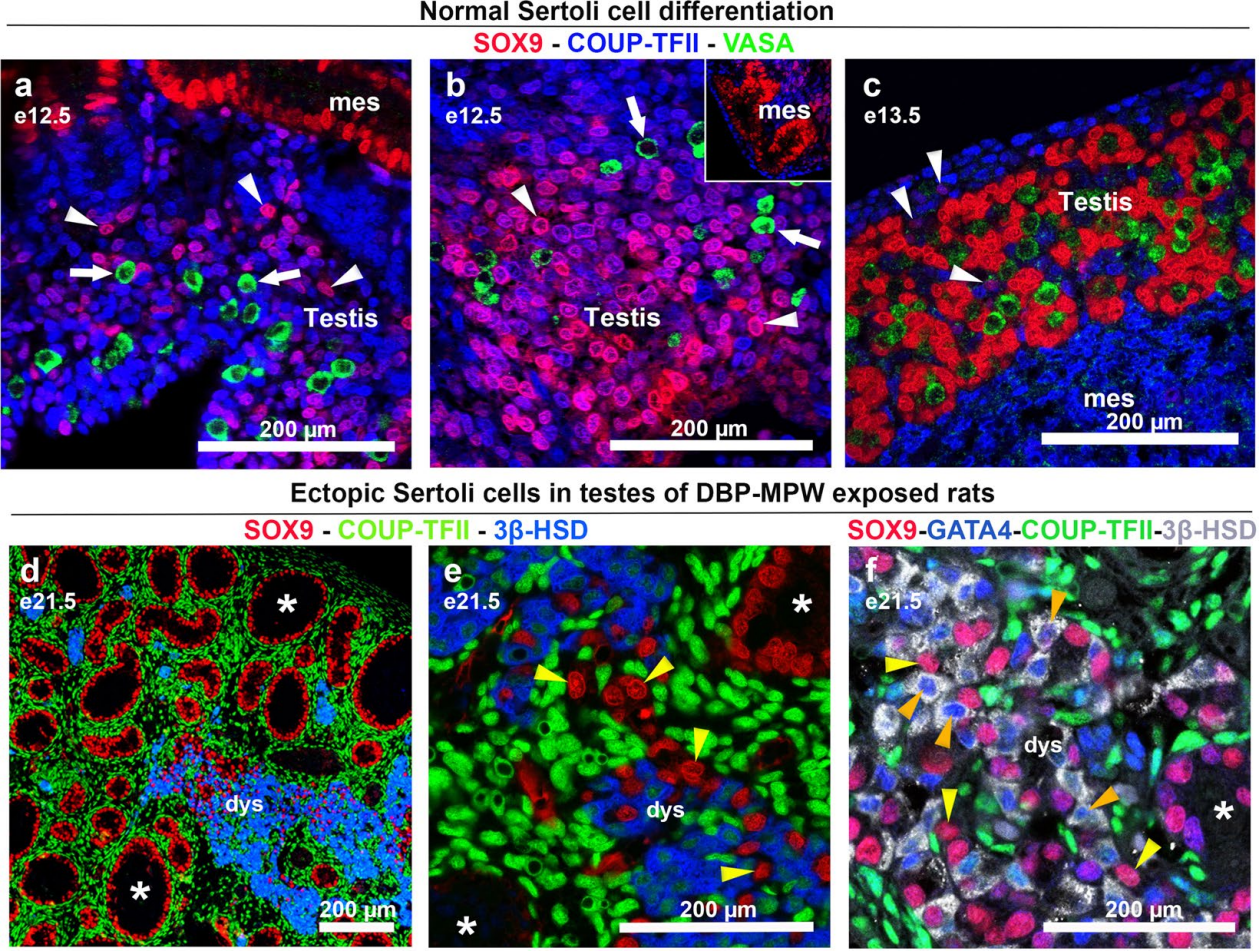
Protein expression pattern of ectopic Sertoli cells at e21.5 in the testes of DBP-MPW animals in comparison to that of Sertoli cells during normal differentiation at e12.5–e13.5. (**a**–**c**) Sections were triple immunostained for SOX9 (red, Sertoli cells), COUP-TFII (blue, mesenchymal cells) and VASA (green, germ cells) whereas (**d**,**e**) were immunostained for SOX9 (red, Sertoli cells), COUP-TFII (green, mesenchymal cells), and 3β-HSD (blue, Leydig cells); Panel f was co-immunostained for SOX9 (red, Sertoli cells), GATA4 (blue, Leydig and Sertoli cells), COUP-TFII (green, mesenchymal cells) and 3β-HSD (grey, Leydig cells). During normal testis development SOX9-immunopositive (Sertoli) cells differentiate from COUP-TFII-immunopositive mesenchymal cells at e12.5 (**a**,**b**: white arrowheads) and when their differentiation is complete at e13.5, the Sertoli cells lose COUP-TFII expression (**c**); germ cells are VASA-immunopositive (green staining, highlighted by white arrows in **a**,**b**). In contrast to the expression pattern during normal testis differentiation, the ectopic Sertoli cells (yellow arrowheads) that appear in late gestation in the testes of DBP-MPW animals never co-express COUP-TFII (**d**,**e**), nor Leydig cell (orange arrowheads) markers (3β-HSD; **f**). *Seminiferous cords; mes = mesonephros; dys = dysgenetic areas.

During normal testis differentiation in our Wistar rats, the Sertoli cells (SOX9+) differentiate at ~e12.5 from undifferentiated mesenchymal cells that express the marker COUP-TFII (Fig. 2a,b). One day later, at e13.5, the Sertoli cells lose their COUP-TFII expression, retaining just SOX9 immunostaining, which we interpret as evidence of completion of their differentiation (Fig. 2c). In contrast, we have been unable to identify any ectopic Sertoli cells that co-express SOX9 + COUP-TFII in the testes of DBP-MPW exposed rats at e19.5–e21.5 which might be expected if they were differentiating de novo outside of the seminiferous cords (Fig. 2d,e). In addition, the ectopic Sertoli cells expressed only the expected differentiated Sertoli cell markers (SOX9, GATA4) comparable to normal intracordal Sertoli cells, and never co-expressed Leydig (3β-HSD) and Sertoli cell markers (Fig. 2f); the latter finding was interpreted as evidence that the ectopic Sertoli cells did not transdifferentiate from fetal Leydig cells (also GATA4-immunopositive) after DBP exposure (Fig. 2f). Based on these observations, we rejected the hypothesis that the DBP-induced ectopic Sertoli cells arise by *de novo* differentiation outside of the seminiferous cords after e19.5.

### Seminiferous cords form normally in the testes of DBP-exposed rats

**Figure 3:**
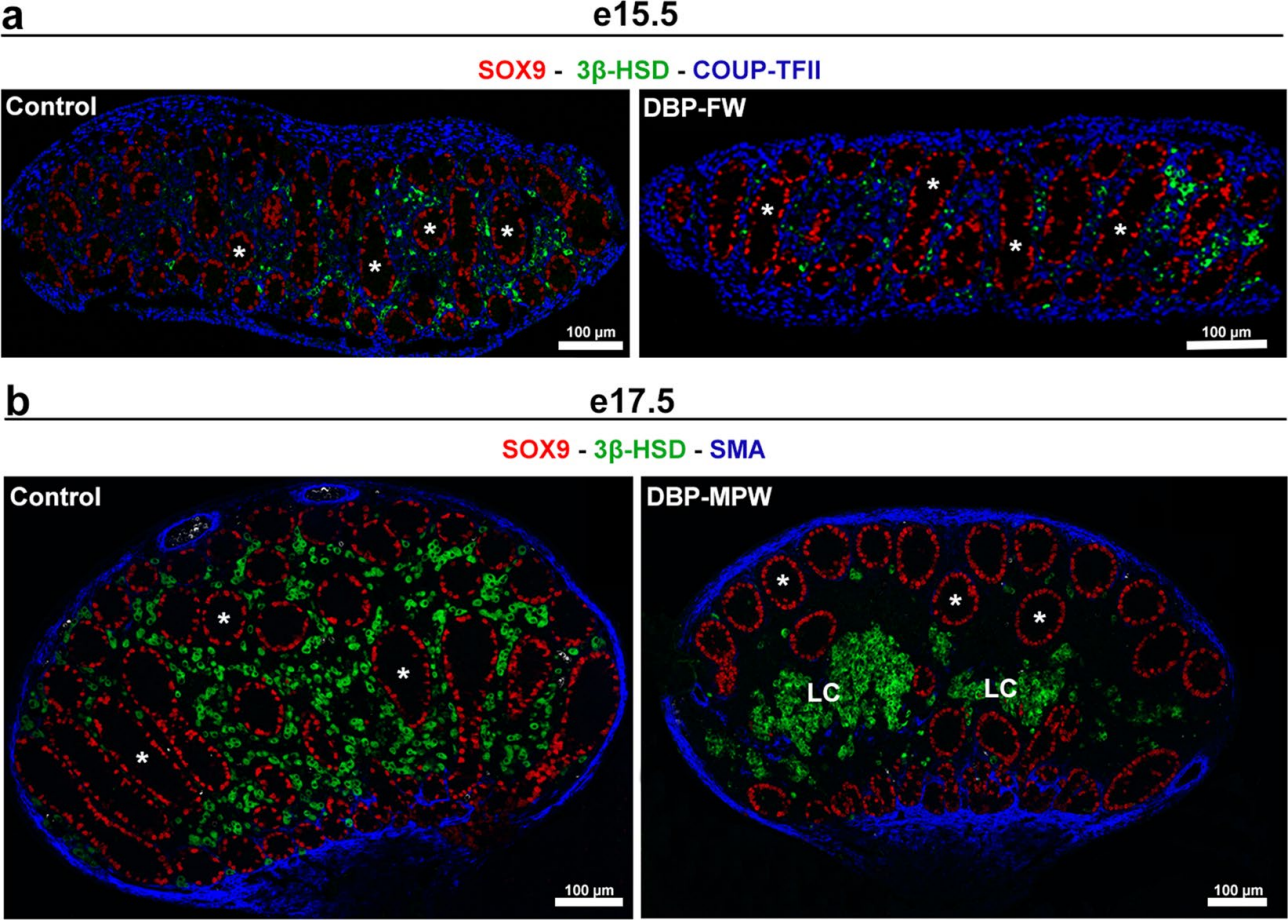
Seminiferous cord formation occurs normally after exposure to DBP during either the FW or the MPW. Sections in (**a**) were triple immunostained for SOX9 (red, Sertoli cells), 3β-HSD (green, Leydig cells) and COUP-TFII (blue, mesenchymal cells) whereas sections in (**b**) were immunostained for SOX9 (red, Sertoli cells), 3β-HSD (green, Leydig cells) and smooth muscle actin (blue). Two days after the beginning of DBP treatment (e15.5, DBP-FW treatment; e17.5, DBP-MPW treatment), the seminiferous cords are normally formed (*) in the testis and only occasional ectopic Sertoli cells are seen, comparable to the situation in controls (see Fig. 4). Note the abnormal central Leydig cell aggregation (LC) at e17.5 in the testis of the DBP-MPW exposed rat, which constitutes the first sign of dysgenesis.

To determine whether DBP exposure in either the FW or the MPW altered normal seminiferous cord formation, we examined whole testis cross sections 2 days after commencement of DBP exposure. When DBP treatment commenced at e13.5 (DBP-FW group), which is the time of normal seminiferous cord formation, the seminiferous cords appeared normally formed when examined 2 days later at e15.5 (DBP-FW; Fig. 3a). Similarly, in DBP-MPW animals, in which treatment had commenced at e15.5 (i.e. after completion of normal seminiferous cord formation), the seminiferous cords appeared normally formed when examined 2 days later at e17.5 (DBP-MPW; Fig. 3b). No focal dysgenetic areas, malformed cords or the presence of supranormal numbers of ectopic Sertoli and germ cells in the interstitial compartment were seen, although at e17.5 abnormal aggregation of the fetal Leydig cells (3β-HSD+) was already evident, which we class as the first visible sign of focal dysgenesis (Fig. 3b).

### DBP-MPW exposure induces rupture of seminiferous cords

**Figure 4:**
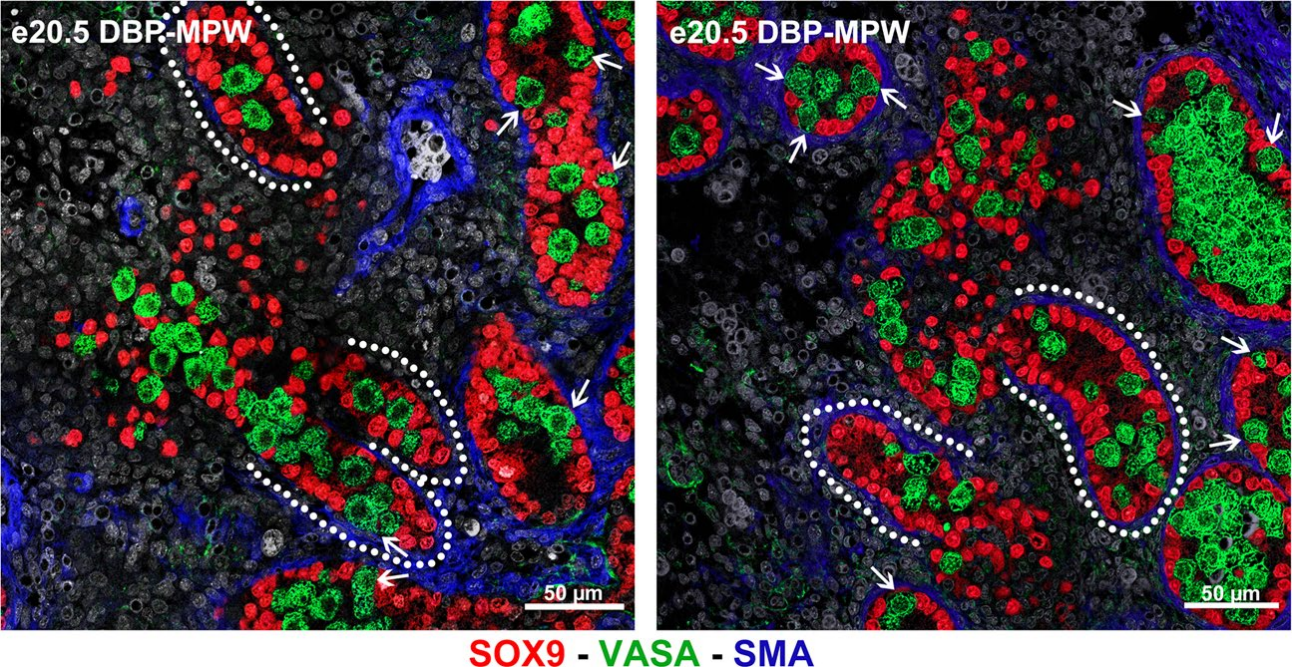
Evidence for seminiferous cord rupture as the source of focal dysgenetic areas in DBP-MPW animals. At e20.5, several examples of seminiferous cords in the process of breaking up could be found in testes of DBP-MPW exposed animals, delineated by the white dotted lines. Rupture can be seen to be releasing Sertoli (red) and germ cells (green) from the cords into the interstitial compartment. Sections were triple immunostained for SOX9 (red, Sertoli cells), VASA (green, germ cells), and smooth muscle actin (blue, peritubular myoid cells, blood vessels). Note the lack of SMA immunoexpression at the point where cord rupture appears to be happening, whereas SMA immunoexpression is present at the intact end of the seminiferous cords. Arrows = germ cells that have migrated to the basal lamina.

The second hypothesis that we tested was that DBP-induction of ectopic Sertoli and germ cells resulted from the rupture of normally formed seminiferous cords beyond e18.5. Thus, testis cross-sections from DBP-MPW animals were qualitatively analysed at e19.5, e20.5 and e21.5. At e20.5, we found examples where seminiferous cords appeared to be breaking up and releasing their contents (Sertoli and germ cells) to the interstitial compartment (Fig. 4). These cords had one end normally formed, with a normal SMA-immunopositive peritubular myoid cell layer, while the other end lacked SMA staining and appeared to be open, probably at the point of rupture. In addition, some SMA ‘patches’ could be seen among the ectopic Sertoli and germ cells in the dysgenetic area immediately adjacent to the ruptured cord end. This rupture of the cords could be seen in several cross sections, obtained from different animals and litters.

### DBP-MPW fetuses exhibit more extensive dysgenesis than DBP-FW fetuses

**Figure 5:**
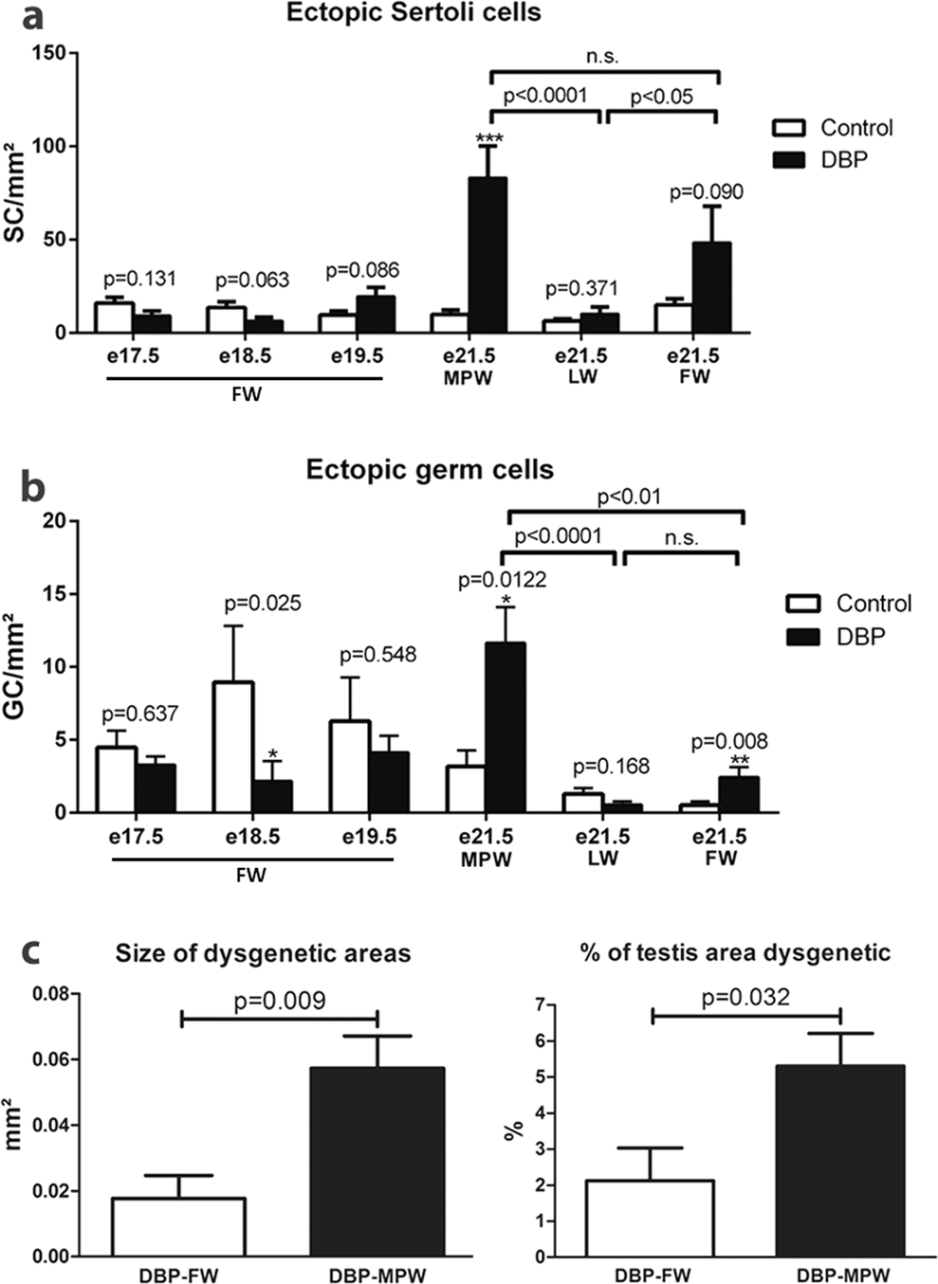
DBP-induction of testicular dysgenesis in relation to the time-window of DBP exposure. Dysgenesis was measured by the number of ectopic Sertoli (SC; **a**) and germ cells (GC; **b**) per mm² testis cross-section between e17.5 and e21.5, or at e21.5 (**c**) as the average size of dysgenetic areas per testis cross-section or as the % of testis cross-sectional area that was dysgenetic. Note that whichever measure of dysgenesis is used, it was always significantly more extensive in testes of DBP-MPW animals when compared to DBP-FW animals, whereas DBP-LW animals did not exhibit dysgenesis. Values are means ± SEM for 5–12 animals per group. Data were log-transformed to normalise variances and analyzed by Student’s t-test, when comparison involved only two groups (control vs. DBP-exposed or DBP-FW vs. DBP-MPW); and by one-way ANOVA followed by Tukey’s post-hoc test, when appropriate. *p < 0.05, **p < 0.01, ***p < 0.001, n.s. = not significant.

Quantitative analysis of dysgenetic areas was performed in order to confirm the impression that restriction of DBP exposure to the MPW resulted in more extensive dysgenesis than if the DBP treatment encompassed a longer period (DBP-FW) that included the MPW. No difference was seen in the number of ectopic Sertoli cells per mm² of testis between control and DBP-MPW or DBP-FW fetuses during the period from e17.5–e19.5 (Fig. 5a). However, at e21.5, a significant increase in numbers of ectopic Sertoli cells/mm² in the DBP-MPW group was evident when compared to controls, and a similar trend, although not statistically significant, was observed for the e21.5 DBP-FW group (Fig. 5a). Both DBP-MPW and DBP-FW groups exhibited significantly more ectopic Sertoli cells per mm² at e21.5 than did the DBP-LW group (in which dysgenesis was not present) (Fig. 5a). At e21.5, both DBP-MPW and DBP-FW groups showed significantly higher numbers of ectopic germ cells when compared to controls. However, the DBP-MPW group had significantly more ectopic germ cells at e21.5 than did the DBP-FW or DBP-LW groups, but there was no significant difference between the latter two groups (Fig. 5b). Consistent with the higher numbers of ectopic Sertoli and germ cells in the DBP-MPW group, the average size of focal dysgenetic areas and the overall percentage of testis area occupied by dysgenesis, were both significantly greater than in the testes of DBP-FW animals (Fig. 5c). Thus, whichever way we analysed dysgenesis, we found that the animals exposed to DBP just during the MPW (e15.5–e18.5) exhibited more pronounced dysgenesis than did animals exposed to DBP before, during and after the MPW (i.e. FW; e13.5–e20.5).

### Effect of DBP exposure on functional markers of peritubular myoid cells and the relationship to dysgenesis

**Figure 6:**
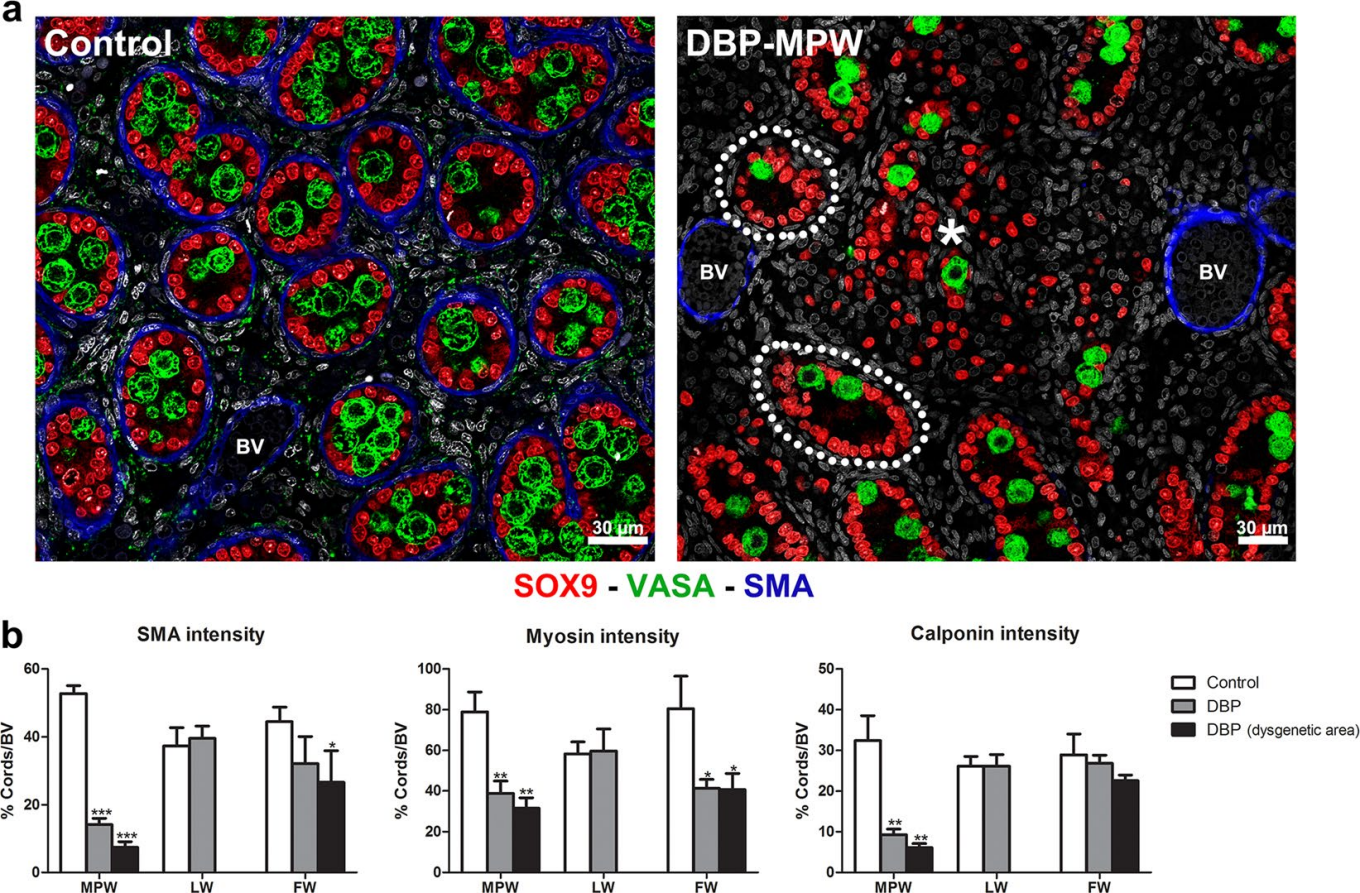
Evidence for functional impairement of peritubular myoid cells around the seminiferous cords in relation to dysgenetic areas in testes of DBP-exposed fetuses. (**a**) sections triple immunostained for SOX9 (red, Sertoli cells), VASA (green, germ cells), and smooth muscle actin (SMA, blue, peritubular myoid cells and some blood vessels, BV). While the control section shows normal SMA immunostaining in peritubular myoid cell around the seminiferous cords and in smooth muscle cells of blood vessels (BV), in the DBP-MPW exposed fetuses the seminiferous cords close to a dysgenetic area (*) lack SMA immunostaining (examples circled by a white dotted line), although SMA is still expressed normally in adjacent BV. (**b**) Quantitative analysis of the immunoexpression intensity for three different peritubular myoid cell functional markers (SMA, myosin, calponin) in the seminiferous cords, normalized to BV immunoexpression of the same protein. Note that in the DBP-MPW and DBP-FW groups, immunoexpression of each marker in peritubular myoid cells was reduced in relation to BV immunoexpression intensity (except for calponin in DBP-FW), while the DBP-LW group showed immunoexpression intensity comparable to controls. Values are means ± SEM for 5–6 animals per group. Data were analysed by one-way ANOVA followed by Tukey’s post hoc test. *p < 0.05, **p < 0.01, ***p < 0.001.

SMA, myosin and calponin are normally expressed in the peritubular myoid cells around the seminiferous cords in the e21.5 fetal rat testis, and are also expressed in smooth muscle cells around larger blood vessels in the interstitium (Fig. 6a,b and Supplementary Fig. S2). The immunoexpression intensity of these markers in peritubular myoid cells was measured and normalized to the intensity of immunoexpression of the same marker in the blood vessels in the same testis cross section. The DBP-LW group, that did not have any dysgenetic areas, showed normal immunoexpression of the three protein markers, similar to controls (Fig. 6b; Supplementary Fig. S2). In the DBP-MPW group, the intensity of immunoexpression of all three markers was reduced in peritubular myoid cells around all seminiferous cords present in the cross sections, and the magnitude of reduction was significantly more pronounced around cords bordering a dysgenetic area (Fig. 6a,b; Supplementary Fig. S2). The DBP-FW group, which also showed focal dysgenetic areas, exhibited a significant reduction in myosin expression around seminiferous cords and in those close to dysgenetic areas whereas SMA immunoexpression was only reduced around cords bordering dysgenetic areas (Fig. 6b). Calponin imunoexpression in peritubular myoid cells did not show a significant difference between the DBP-FW and control groups (Fig. 6b; Supplementary Fig. S2).

### Differential effects of DBP exposure on germ cell migration to the basal lamina

**Figure 7:**
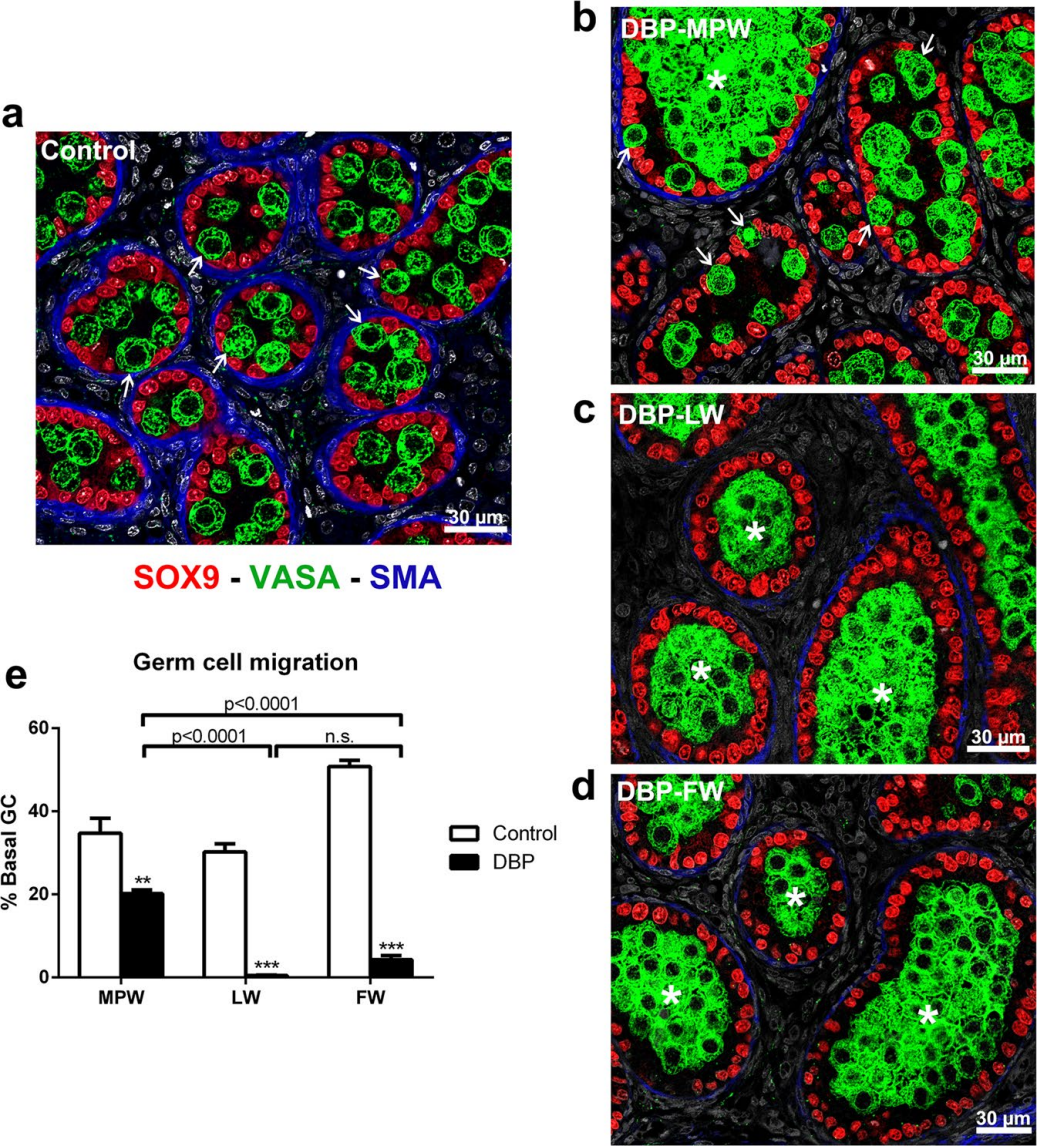
Quantitative analysis of the effect of DBP exposure on germ cell migration to the basal lamina in the e21.5 rat testis. Sections were triple immunostained for SOX9 (red, Sertoli cells), VASA (green, germ cells), and smooth muscle actin (blue, peritubular myoid cells and some blood vessels). In (**a**), note that in controls at this age the germ cells are migrating outwards to the basal lamina of the cords (arrows). In (**b–d**), after DBP exposure germ cells are clustered in the centre of the cords (asterisks), but this is less extensive in the DBP-MPW group (**b**) in which some germ cells still show migration to the basal lamina (arrows), whereas this is rarely seen in the DBP-LW (**c**) and DBP-FW (**d**) groups. (**e**) Quantitative analysis of the percentage of basally migrated germ cells (GC) in the different treatment groups. Values are means ± SEM for 5–6 animals per group. Data were analyzed by Student’s t-test, when comparison involved only two groups (control vs. DBP-exposed); and by one-way ANOVA followed by Tukey’s post-hoc test, when appropriate. **p < 0.01, ***p < 0.001, n.s. = not significant.

During normal development of the testis in rats, at around e21.5 the germ cells are already migrating outwards to gain contact with the basal lamina, where they will differentiate into spermatogonia (Fig. 7a). DBP treatment causes germ cell aggregation in the centre of seminiferous cords, reducing the percentage of germ cells that migrate to the basal lamina, but this is influenced by the timing of DBP exposure (Fig. 7b–e). Thus, DBP exposure during the LW (i.e. DBP-LW and DBP-FW groups) resulted in near complete prevention of germ cell migration to the basal lamina, whereas in the DBP-MPW group, significantly more germ cells migrated to the basal lamina, coincident with a notable reduction in germ cell aggregation (Fig. 7b–e).

### Presence of focal dysgenetic areas in the postnatal testes of DBP-MPW animals

**Figure 8:**
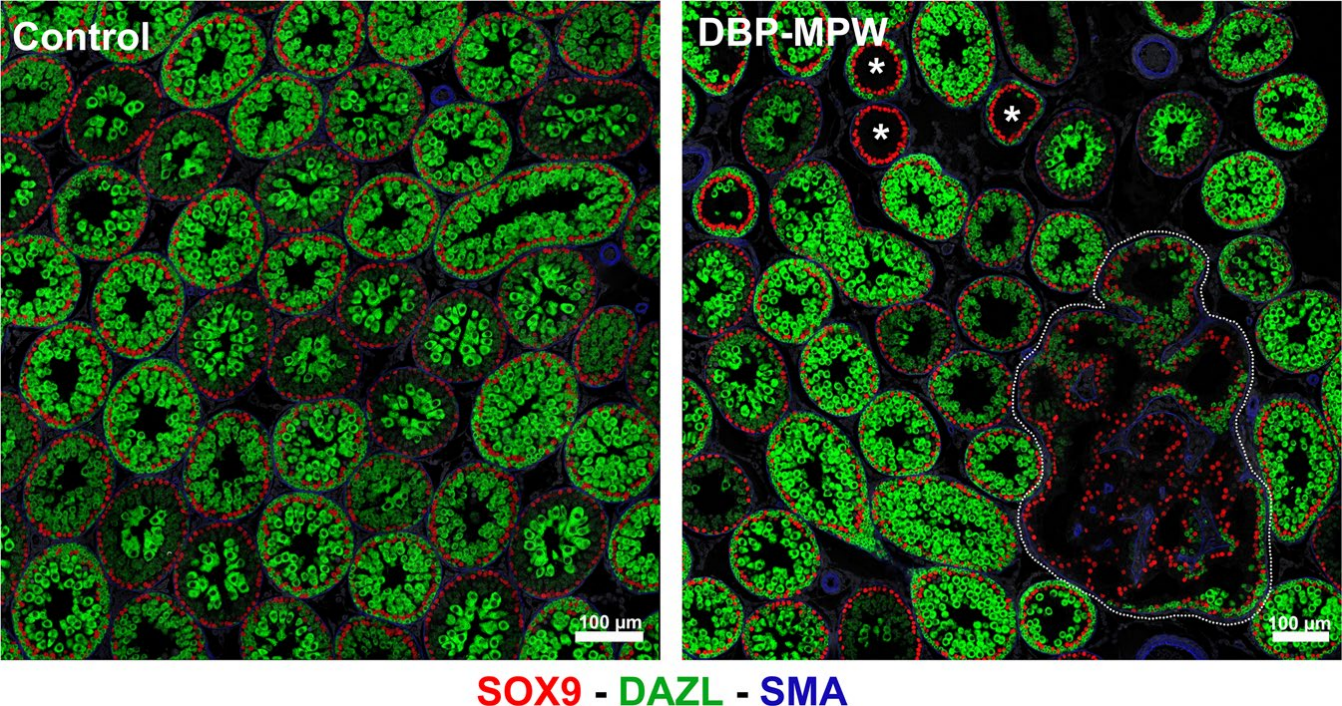
Identification of focal dysgenetic areas in the testes of DBP-MPW exposed animals at postnatal day 25 (Pnd25). Sections were triple immunostained for SOX9 (red, Sertoli cells), DAZL (green, germ cells), and smooth muscle actin (blue, peritubular myoid cells and some blood vessels). In the representative DBP-MPW exposed animal, a focal dysgenetic area (circled by the white dotted line), with malformed tubules and abnormal cell composition, can be seen among the normal testicular parenchyma; tubules with impaired spermatogenesis are also present (asterisks). Controls showed none of these features.

The focal dysgenetic areas evident in the fetal testis become transformed after birth into focal areas with abnormal/anastomotic seminiferous tubules, as is illustrated in Fig. 8 for a postnatal day (Pnd) 25 rat that had been exposed to DBP during the MPW. In this figure, a dysgenetic area can be seen among the normal testicular parenchyma, with abnormal and disorganized seminiferous tubules, and some tubules also presenting with deficient spermatogenesis. We have shown previously that this phenotype persists through to adulthood^12, 18, 24^.

## Discussion

The testicular dysgenesis syndrome (TDS) hypothesis has become the main focus for studies into the origins of the commonest male reproductive disorders in newborn boys and young men^1, 7, 11^. This hypothesis argues that faults in the initial ‘set-up’ of the testis leads to somatic cell dysfunction which leads secondarily to TDS disorders. The present findings, in an established rat model for TDS^12, 13, 23, 37^, add a new dimension to this thinking, as they demonstrate that induction of focal dysgenesis in the rat testis, which is intimately associated with occurrence of later TDS disorders^12, 15–17, 33^, is induced after normal testis cell differentiation and organisation have occurred. Thus, our finding challenges current paradigms about testis development and TDS, by demonstrating that normally formed seminiferous cords can be disassembled after exposure to DBP; of critical importance is that this disassembly is only inducible after DBP exposure in the MPW, which is the fetal time-window within which androgen deficiency leads to TDS disorders.

Our previous studies have shown that focal dysgenetic areas in the adult testes of rats exposed in fetal life to DBP, probably originate from focal areas of ectopic Sertoli cells within the fetal testis^12, 18, 23, 24, 38^. The present study has used cell-specific immunostaining to elucidate the origin of these ectopic Sertoli cells. Using DBP exposure during three different fetal time-windows, we have shown that the ectopic Sertoli cells originate in late gestation (e19.5 onwards), long after seminiferous cord formation, and only occur when there has been exposure to DBP during the MPW (e15.5–e18.5); they are not induced by DBP exposure after e18.5 (DBP-LW). Thus, previous hypotheses that attributed the presence of ectopic Sertoli cells to malformation of the seminiferous cords^12, 21, 22^ could have an alternative explanation, based on the present findings.

We considered two possible mechanisms of origin for the ectopic Sertoli cells. The first, *de novo* differentiation of these Sertoli cells in the interstitial compartment, was addressed by a comparative analysis of SOX9 and COUP-TFII immunoexpression in the ectopic Sertoli cells. During normal testis differentiation, the Sertoli cells differentiate (e12.5–e13.5) from a population of mesenchymal cells in the genital ridge, which express COUP-TFII (present study); once Sertoli cell differentiation is complete, COUP-TFII is switched off. Fetal Leydig cells subsequently also differentiate from mesenchymal cells that express COUP-TFII^39^. However, when we studied the protein expression pattern of ectopic Sertoli cells in the e19.5–e21.5 testes of DBP-MPW exposed fetuses, we failed to find any Sertoli cells that co-expressed COUP-TFII and SOX9, nor could we find any evidence that the ectopic Sertoli cells co-expressed Leydig cell-specific markers (3β-HSD), that might be indicative of Sertoli cell transdifferentiation from fetal Leydig cells. From these observations, we considered it unlikely that the ectopic Sertoli cells originate by *de novo* differentiation. This led us to investigate the second possibility, that the ectopic Sertoli cells originate from the rupture of normally formed seminiferous cords.

Most studies that have investigated the effects of DBP exposure on the fetal rat testis have used daily treatment of the pregnant mother from e13.5 (when seminiferous cord formation is in progress) until e21.5, which we refer to as ‘full window’ DBP treatment (DBP-FW). We show presently that seminiferous cord formation occurs normally in DBP-FW fetuses when examined at e15.5. Similarly, in DBP-MPW animals, in which the DBP treatment commences after completion of cord formation, seminiferous cords remained intact until at least e17.5. Moreover, the number of ectopic Sertoli and germ cells in DBP-FW animals at e17.5–e19.5 remained comparable to that found in controls; we presume that the ‘normal’ ectopic Sertoli and germ cells found in controls are cells that failed to get incorporated into seminiferous cords, as their numbers (in controls) remained fairly constant throughout the period e17.5–e21.5. However, by e21.5 in DBP-MPW animals, the number of ectopic Sertoli cells increased considerably above background (control) levels and was paralleled by an increase in ectopic germ cells. DBP-FW animals also showed an increase in ectopic Sertoli and germ cells at e21.5 (not significant for Sertoli cells), but the numbers were significantly less than for DBP-MPW animals. Corresponding with these differences in ectopic cell numbers, both the total area of dysgenesis per testis as well as the size of individual focal dysgenetic areas was also significantly larger in DBP-MPW than in DBP-FW animals. This counter-intuitive finding is discussed further below.

In view of the parallel increase in ectopic Sertoli and germ cells that was evident at e21.5 in DBP-MPW and DBP-FW animals, we considered it likely that these cells originated from rupture of seminiferous cords. Therefore, we searched for such cords on the two days prior to e21.5 in DBP-MPW animals and found several examples of seminiferous cords in the process of rupture at e20.5, as illustrated in Fig. 4. We considered this to be conclusive. However, this observation raised two inter-related questions – first, why did some cords rupture and some not, and second, why was this more severe/extensive in animals exposed to DBP only during the MPW than in those exposed to DBP for a longer period (DBP-FW) that included the MPW. To answer these questions we focussed on the peritubular myoid cells, which constitute the outer boundary of the seminiferous cords/tubules and which, together with Sertoli cells, are responsible for secreting the basal lamina that surrounds and physically supports the cords^40^.

Peritubular myoid cells express cytoskeletal proteins similar to other smooth muscle cells, including myosin (Myh11), desmin/vimentin, alpha-actin, smooth muscle actin (SMA) and calponin (a functional marker of peritubular myoid cell contractility), and expression of these proteins is considered to be indicative of normal differentiation/function of these cells^41–43^. We undertook quantitative analysis of the immunoexpression of three of these proteins (SMA, myosin and calponin) by peritubular myoid cells, and showed that their expression was significantly reduced in testes of DBP-MPW animals, an effect that was especially pronounced in cords bordering focal dysgenetic areas. DBP-FW animals also showed significant reductions in immunoexpression of SMA and myosin in peritubular myoid cells, but not of calponin, although in general these effects were less pronounced than those seen in DBP-MPW animals. In DBP-LW animals, no change in immunoexpression of any of the three proteins in peritubular myoid cells was found. These results pointed to a clear association between reduced immunoexpression of SMA, calponin and probably myosin and the occurrence of focal dysgenetic areas. Indeed, the ‘ruptured’ seminiferous cords found in e20.5 DBP-MPW animals shared similar characteristics: one end of the cord seemed normally formed but the other end lacked peritubular myoid cell-SMA staining at the point where the rupture was happening, with some SMA ‘patches’ among the ectopic cells, as if the peritubular myoid cells had been dispersed. To our knowledge, this is the first time that the rupture of previously normal seminiferous cords has been reported in the fetal testis. Furthermore, the association between greater magnitude of reduction in peritubular myoid cell protein immunoexpression with more extensive dysgenesis could be interpreted as evidence for cause and effect. However, from observation of the germ cells in the different DBP treatment groups, another potential determinant of the severity of dysgenesis was identified which may explain why dysgenesis is more severe in DBP-MPW animals.

Despite not presenting with focal dysgenetic areas, DBP-LW animals exhibited abnormal aggregation of the germ cells in the centre of the seminiferous cords at e21.5, an effect that has been widely reported previously in DBP-exposed fetuses^44–46^; the germ cell aggregation results from the withdrawal of Sertoli cell cytoplasmic contacts with the germ cells^46^. This aggregation almost completely prevents the migration of germ cells outwards to the basal lamina, a process that is normally in progress at e21.5 in the rat testis^47, 48^. The DBP-FW group also exhibited germ cell aggregation and failure of migration comparable to that in the DBP-LW group, consistent with the germ cell aggregation being an effect induced primarily in late gestation^44–46^. In keeping with this, animals from the DBP-MPW group had only occasional germ cell aggregation within the cords, and consequently a much higher percentage of germ cells had migrated normally down to the basal lamina, although still fewer than in controls. On the assumption that there is reduced functionality of the peritubular myoid cells, as evidenced by SMA, calponin and myosin expression, we hypothesize that the far greater number of germ cells migrating to the basal lamina in DBP-MPW animals, compared with DBP-FW animals, results in breeching of a presumptively weakened basal lamina. This would explain why a much higher number of ectopic Sertoli and germ cells is observed in the dysgenetic areas after DBP exposure within the MPW compared with DBP-FW animals.

The present study reinforces the critical importance of the MPW for normal testis development/function, as it demonstrates that only exposure to DBP specifically within the MPW results in focal dysgenesis, even though this dysgenesis does not become manifest histologically until after the MPW and after cessation of DBP exposure. Focal dysgenesis is closely correlated with reduced androgen production and action within the MPW in DBP-exposed fetuses^23, 34^, which presumably explains why occurrence of focal dysgenesis in the adult testes of DBP-exposed animals is closely linked to occurrence and severity of male reproductive disorders that result from fetal androgen deficiency in the MPW^33, 34, 36^. Moreover, the phenotypic presentation of focal dysgenesis in postnatal life, as shown presently in early puberty (Pnd25), or in adulthood^16, 18, 49^, after DBP-MPW exposure, shows similarities to focal dysgenetic changes seen in the testes of presumptive human TDS cases^3–5, 8^. Thus, the present new findings might be relevant to understanding how and when dysgenesis arises in the testes of human TDS cases, assuming that the mechanisms that underlie DBP-induced dysgenesis in the fetal rat testis have relevance to the human.

## Material and Methods

### Animals, treatments, sample collection and processing

All aspects of animal housing, management and treatment conformed to UK home office guidelines and all experiments were conducted under their specific project licence approval (RMS-PPL 60/4564); all experimental protocols were approved by the University of Edinburgh animal welfare and ethical review body as part of the project licence application. Rats were fed a soy-free breeding diet (RM3(E); SDS, Dundee, Scotland). Housing conditions were carefully controlled, with lights on at 07:00, off at 19:00, temperature at 19–21 °C, GOLD shavings and LITASPEN standard bedding. Randomly allocated time-mated females received either vehicle control treatment or 750 mg/kg dibutyl phthalate (DBP; 99% pure; Sigma-Aldrich) in 1 mL/kg corn oil, daily by oral gavage, during the following treatment windows:

- FW (Full Window) = from embryonic day (e)13.5 to the day before termination (eg. e13.5 to e20.5, termination e21.5).
- MPW (Masculinisation Programming Window) = from e15.5 to e18.5.
- LW (Late Window) = from e19.5 to e20.5.

All treatments were administered in a single animal facility at the University of Edinburgh. Male offspring were sampled on e17.5, e18.5, e19.5, e21.5 from the FW treatment; e19.5, e20.5, e21.5 and postnatal day (Pnd) 25 from the MPW treatment; and e21.5 from the LW treatment; control male fetuses were also sampled at e12.5 and e13.5, to follow normal testis development. Time points were chosen to reflect the period before, during, and after the appearance of DBP-induced dysgenesis. Pregnant dams were killed by CO_2_ inhalation followed by cervical dislocation. Fetuses were removed, decapitated and placed in ice-cold phosphate-buffered saline (PBS; Sigma-Aldrich). Postnatal pups were housed with their natural mothers from birth and killed by cervical dislocation. Fetuses and pups were transported immediately to the laboratory where testes were removed by microdissection, fixed for 1 hour in Bouin’s fixative (6 hours for Pnd25 testes) then transferred to 70% ethanol and processed into paraffin blocks using standard methods. We used 5–12 animals from 3–6 litters per treatment group, all experiments including animals from each of these litters. Testes were serially sectioned at 5 µm, and 3 sections were used per animal for each analysis, corresponding to approximately 25, 50, and 75% intervals through the serially sectioned testis.

### Immunofluorescence

Triple immunostaining was used for co-immunolocalization of the different cell types, using specific antibodies listed in Table 1. All incubations were carried out in a humidity box (Fisher Scientific) and the slides were washed in between all incubation steps in TBS (2 × 5 min). Sections were dewaxed and rehydrated using standard procedures, followed by a peroxidase block in 3% H_2_O_2_ in methanol for 30 min. The sections were blocked with normal chicken serum (NChS; Biosera) diluted 1:5 in TBS containing 5% BSA (NChS/TBS/BSA), followed by incubation with the first primary antibody, diluted in NChS/TBS/BSA, overnight at 4 °C. The next day, sections were incubated with the relevant peroxidase-conjugated secondary antibody, diluted in NChS/TBS/BSA for 30 min at room temperature (RT), followed by incubation with Tyramide (TSA-Plus Cyanine 3 System; Perkin Elmer Life Sciences) diluted 1:50 in its buffer, for 10 min. Before the next primary antibody dilution was added, the sections were subjected to antigen retrieval by boiling in a pressure cooker in 0.01 mol/L citrate buffer (pH 6.0) for 5 min. This was followed by another block in NChS/TBS/BSA and overnight incubation at 4 °C with the next primary antibody, diluted in NChS/TBS/BSA. On the third day, slides were incubated with the relevant peroxidase-conjugated secondary antibody diluted in NChS/TBS/BSA for 30 min at RT, followed by incubation with Tyramide (TSA-Plus Cyanine 5 System; Perkin Elmer Life Sciences) diluted 1:50 in its buffer, for 10 min. Sections were again blocked against peroxidase in 3% H_2_O_2_ in TBS plus 0.01% Tween-20 (Sigma-Aldrich) for 20 min followed by blocking in NChS/TBS/BSA and incubation with the third primary antibody diluted in NChS/TBS/BSA overnight at 4 °C. Sections were then incubated with the relevant peroxidase-conjugated secondary antibody diluted in NChS/TBS/BSA for 30 min at RT, followed by incubation with Tyramide (TSA-Plus Fluorescein System; Perkin Elmer Life Sciences) diluted 1:50 in its buffer, for 10 min. Nuclear counterstain (DAPI, Sigma-Aldrich) was diluted 1:500 in TBS and incubated for 10 min. In case a quadruple staining was needed, sections were blocked against peroxidase after the third Tyramide step, as described above, followed by blocking in NChS/TBS/BSA and incubation with the fourth primary antibody, overnight at 4 °C. A biotinylated secondary antibody was added on the following day, diluted 1:500 in NChS/TBS/BSA, followed by detection with streptavidin conjugated to Alexa Fluor 405 (ThermoFisher Scientific; s32351), diluted 1:200 at room temperature. Finally, slides were mounted with Permafluor (Thermo Scientific) and fluorescent images captured using a Zeiss LSM 780 confocal laser microscope, generating high resolution tiled confocal scanning laser microscopy images of the complete fetal testis sections.

**Table 1:** Antibodies used for immunofluorescence.

Antibody	Source	Species	Dilution
SOX9	Chemicon International (AB5535)	Rabbit	1:5000
COUP-TFII	Perseus Proteomics Inc. (PP-H7147-00)	Mouse	1:1000
VASA	Abcam (ab13840)	Rabbit	1:400
3β-HSD	Santa Cruz Biotechnology (sc30820)	Goat	1:200
GATA4	Santa Cruz Biotechnology (sc25310)	Mouse	1:500
DAZL	AbD Serotec (MCA2336)	Mouse	1:1000
SMA	Sigma (A2547)	Mouse	1:10000
Calponin	Abcam (ab46794)	Rabbit	1:1000
Myosin	Abcam (ab33219)	Rabbit	1:100
ChARP	Santa Cruz Biotechnology (sc2963)	Chicken	1:200
ChAMP	Santa Cruz Biotechnology (sc2962)	Chicken	1:200

Abbreviations: SMA, smooth muscle actin; ChARP, chicken anti-rabbit peroxidase; ChAMP, chicken anti-mouse peroxidase.

### Sertoli cell differentiation and seminiferous cord formation

At e12.5 and e13.5, control testis sections were immunostained for SOX9 - COUP-TFII - VASA and analysed to follow normal Sertoli cell differentiation. At e21.5, testes from DBP-exposed fetuses were stained for SOX9 - COUP-TFII - 3β-HSD or for SOX9 - GATA4 - COUP-TFII - 3β-HSD in order to investigate if the ectopic Sertoli cells arise from *de novo* differentiation outside of the seminiferous cords, by comparison to the normal Sertoli cell differentiation at e12.5–e13.5 and by comparing the expression pattern shown by the other key somatic cell, Leydig cells.

The normality of seminiferous cord formation was analysed at e15.5 and e17.5 after DBP exposure starting at e13.5 (DBP-FW) or at e15.5 (DBP-MPW), respectively. Sections were triple immunostained for SOX9 - COUP-TFII - 3β-HSD or for SOX9 - 3β-HSD - SMA in order to visualize the seminiferous cord structure and the possible existence of focal dysgenetic areas.

### Rupture of already-formed seminiferous cords

SOX9 – VASA - SMA triple immunostaining was performed on testis sections from DBP-MPW fetuses at e19.5, e20.5 and e21.5, as described above. Whole testis images were captured and screened carefully to identify examples of seminiferous cords that appeared to have ruptured, releasing Sertoli and germ cells to the interstitial compartment.

### Incidence, size and severity of dysgenetic areas

Whole testis sections were analysed after staining for SOX9 - VASA - SMA. The testis cross sectional area was measured using ZEN 2.3 blue edition software (Carl Zeiss Microscopy GmbH, 2011). The presence of dysgenetic areas, containing Sertoli cells (SOX9-immunopositive) and germ cells (VASA-immunopositive), outside of seminiferous cords (delimited by SMA – immunopositive peritubular myoid cells) was recorded and the number of each type of ectopic cell was counted. Total testis area was measured, by manually delineating the borders of the cross sections with the software tool. When present, the size of the dysgenetic area was also measured, being manually defined as the whole area containing ectopic cells. The number of ectopic Sertoli and germ cells per mm² of testis area was then calculated, as well as the percentage of the testis area that was occupied by dysgenetic regions, determined by the ratio of the dysgenetic area and the total testis area ×100.

### Evaluation of peritubular myoid cell function

The intensity of immunoexpression of SMA, calponin and myosin in peritubular myoid cells that normally surround the outside of seminiferous cords was quantified using ZEN 2.3 blue edition software, which measures the intensity value of each fluorescent channel in a chosen area. The seminiferous cords were circled individually and the relevant fluorescence value for each cord was recorded. Seminiferous cords bordering dysgenetic areas were separately grouped, but also included in the total measurement. The same immunoexpression intensity analysis was applied to smooth muscle cells demarcating blood vessels within the interstitium of each testis, and these were used for normalization for each individual testis sample. All seminiferous cords and immunopositive blood vessels per whole testis cross-section were quantified, comprising 40–100 seminiferous cords and 5–20 blood vessels for each testis. Thus, the intensity of peritubular myoid cell marker immunoexpression was calculated as the mean intensity value around the seminiferous cord divided by the intensity value around blood vessels x100.

### Germ cell migration

Using whole testis images immunostained for SOX9 – VASA - SMA, the percentage of basally migrated germ cells was analysed. Every intracordal germ cell, identified by VASA-positive staining inside seminiferous cords, was counted and classed either as basal, meaning germ cells that were in contact with the basal lamina, or as central, meaning germ cells that had no contact with the basal lamina. The percentage of basal germ cells was calculated as the ratio of the number of basal germ cells to the total number of germ cells in the testis x100.

### Postnatal testis samples

Testis cross sections from postnatal day (Pnd) 25 DBP-MPW animals were immunostained for SOX9 – DAZL - SMA. Testis confocal images were captured and examined in order to understand how the focal dysgenetic areas observed during fetal life develop after birth.

### Statistics

Values are expressed as means ± SEM. Data were analysed by GraphPad Prism 6 (GraphPad Software Inc.) using either one-way ANOVA followed by the Bonferroni post-test or Student’s t-test, as appropriate. Data was log transformed prior to analysis if the distribution and variance was abnormal.

## Electronic supplementary material

Supplementary Information
