# Publisher Correction: Dibutyl phthalate induced testicular dysgenesis originates after seminiferous cord formation in rats

**DOI:** 10.1038/s41598-019-43786-3

**Published:** 2019-10-28

**Authors:** Nathália L. M. Lara, Sander van den Driesche, Sheila Macpherson, Luiz R. França, Richard M. Sharpe

**Affiliations:** 1 MRC Centre for Reproductive Health, The Queen’s Medical Research Institute, University of Edinburgh, Edinburgh, EH16 4TJ UK; 2 Laboratory of Cellular Biology, Department of Morphology, Federal University of Minas Gerais, 31270-901 Belo Horizonte, MG Brazil; 3 National Institute for Amazonian Research, 69067-375 Manaus, AM Brazil; 4 Present Address: Centre for Integrative Physiology, Biomedical Sciences, Hugh Robson Building, University of Edinburgh, Edinburgh, EH8 9XD UK

Correction to: *Scientific Reports* 10.1038/s41598-017-02684-2, published online 31 May 2017

In the original version of this Article, the author Sander van den Driesche was incorrectly indexed. This error has now been corrected.

