# Supplementary material for: Dibutyl phthalate induced testicular dysgenesis originates after seminiferous cord formation in rats
Source: Sci Rep. 2017 May 31;7:2521. doi: 10.1038/s41598-017-02684-2 (PMC5451485; doi:10.1038/s41598-017-02684-2)

Dibutyl phthalate induced testicular dysgenesis originates after seminiferous cord formation in rats

Nathália LM Lara, Sander van den Driesche, Sheila Macpherson, Luiz R França, Richard M Sharpe.

Supplementary Information

Supplementary Figure S1: Comparative size of focal dysgenetic areas in the e21.5 testis after DBP-MPW or DBP-FW exposure. Sections were triple immunostained for SOX9 (red, Sertoli cells), VASA (green, germ cells), and smooth muscle actin (blue). The focal dysgenetic areas, presenting as ectopic Sertoli (red) and germ (green) cells in the interstitial compartment, are circled by a white dotted line. Note that in the DBP-MPW group (A, B), the dysgenetic areas are larger and contain more ectopic cells than do the corresponding areas in the DBP-FW group (C, D).

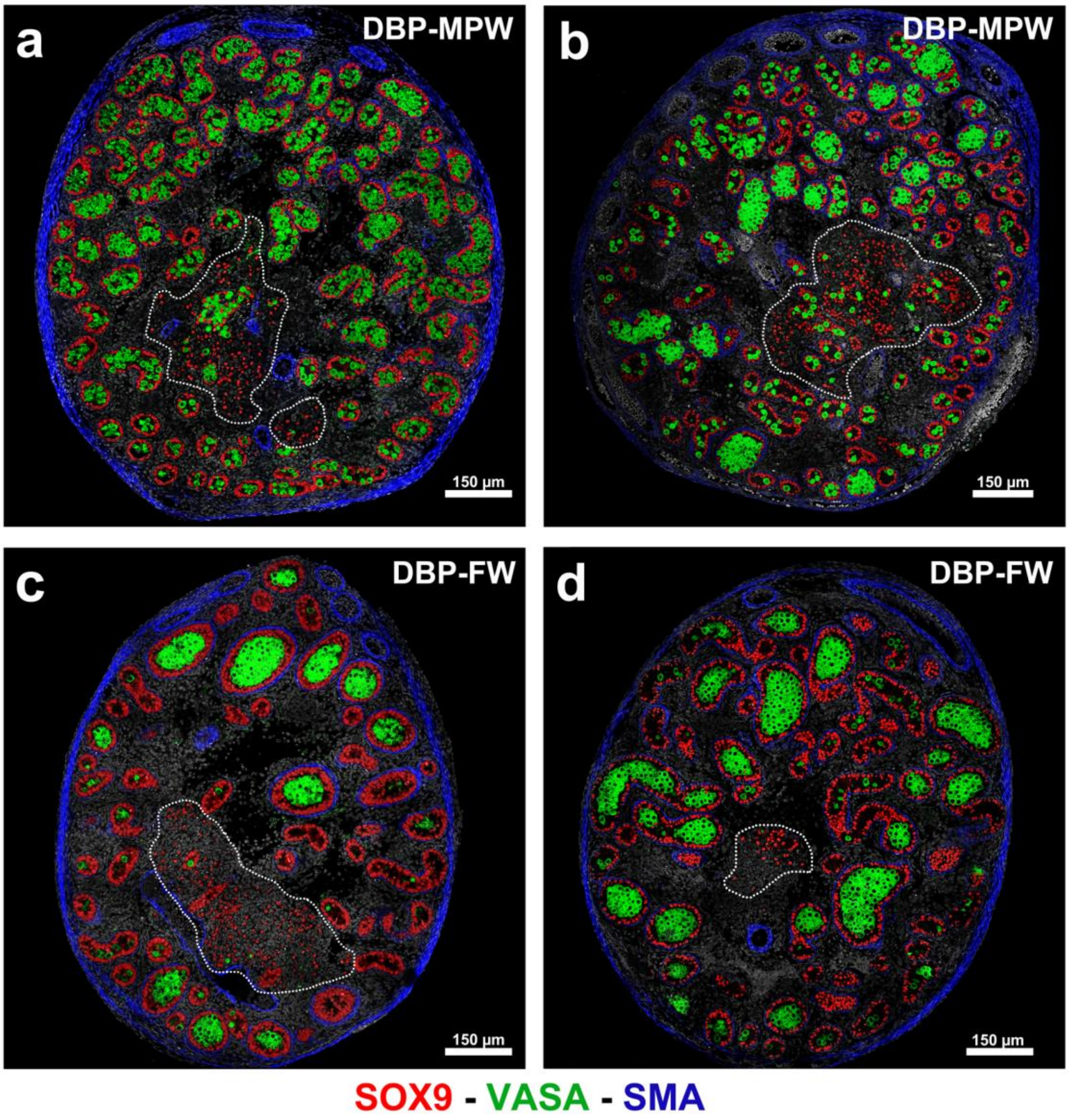

Supplementary Figure S2: Evidence for functional impairement of peritubular myoid cells around the seminiferous cords in testes of DBP-exposed fetuses. Sections immunostained for SOX9 (red, Sertoli cells) and calponin (SMA, blue, peritubular myoid cells and some blood vessels, BV) in A-C, whereas sections in D-F were immunostained for SOX9 (red, Sertoli cells) and myosin (SMA, blue, peritubular myoid cells and some blood vessels). Observe that control sections show normal calponin (A) and myosin (D) immunostaining in peritubular myoid cell around the seminiferous cords and in smooth muscle cells of blood vessels (BV). As expected (see Fig. 6B), in the DBP-MPW exposed fetuses the seminiferous cords close to a dysgenetic area (*) showed reduced calponin (B) and myosin (E) immunostaining (examples circled by a white dotted line), although these markers are still expressed normally in adjacent BV. Regarding the DBP-FW testes, calponin expression (C) was similar to the control section (A), while myosin showed reduced expression around the seminiferous cords (F, white dotted line).

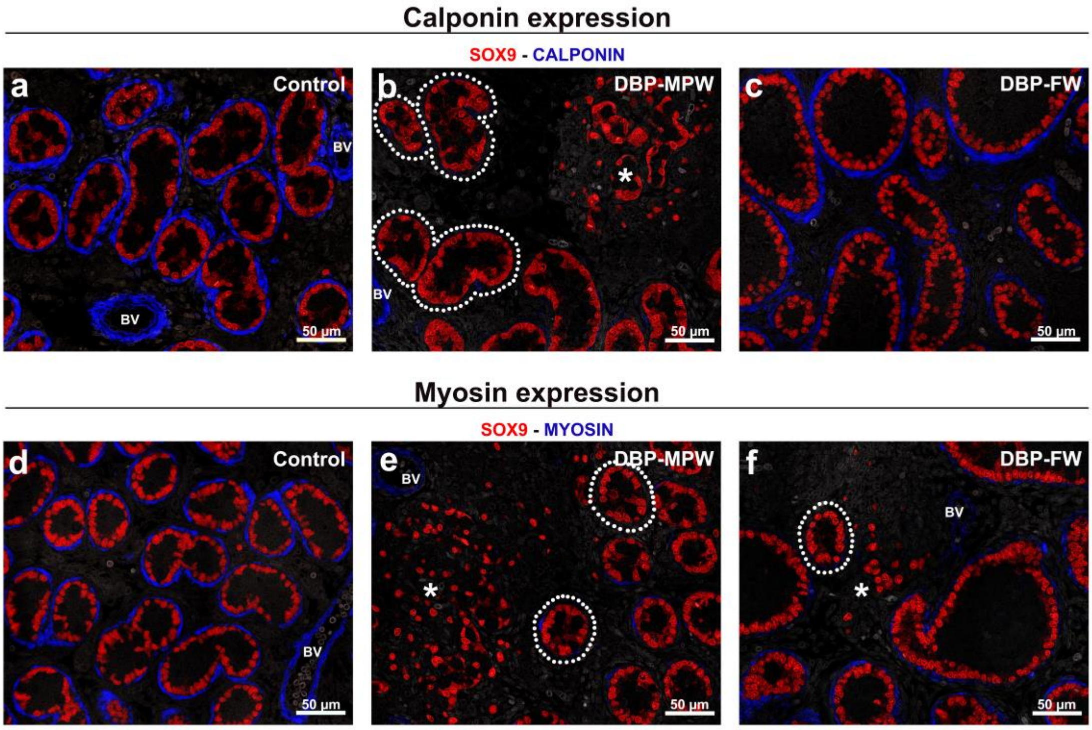

Supplement: Supplementary file 1 — Supplementary Information [file 41598_2017_2684_MOESM1_ESM.pdf]
